# Impact of Propionic Acid on Liver Damage in Rats

**Published:** 2015

**Authors:** Sooad Al- Daihan, Ramesa Shafi Bhat

**Affiliations:** Biochemistry Department, Science College, King Saud University, Riyadh, Saudi Arabia.

**Keywords:** Propionic acid, liver, oxidative stress

## Abstract

Propionic acid (PA) is a short chain fatty acid, a common food preservative and metabolic end product of enteric bacteria in the gut. The present study was undertaken to investigate the effect of PA on liver injury in male rats. Male western albino rats were divided into two groups. The first group served as normal control, the second was treated with PA. The activities of serum hepatospecific markers such as aspartate transaminase, alanine transaminase, and alkaline phosphatase were estimated. Antioxidant status in liver tissues was estimated by determining the level of lipid peroxidation and activities of enzymatic and non-enzymatic antioxidants. Sodium and potassium levels were also measured in liver tissue. PA treatment caused significant changes in all hepatospecific markers. Biochemical analysis of liver homogenates from PA-treated rats showed an increase in oxidative stress markers like lipid peroxidation and lactate dehydrogenase, coupled with a decrease in glutathione, vitamin C and glutathione S- transferase. However, PA exposure caused no change in sodium and potassium levels in liver tissue. Our study demonstrated that PA persuade hepatic damage in rats.

Propionic acid (PA) is a naturally occurring carboxylic acid. This dietary short-chain fatty acid occurs naturally in milk, dairy products such as yogurt and cheese (1, 2). PA is mainly produced by the fermentation of indigested food by the microbiota in the colon, but can reach the blood compartment and the adipose tissue, where it reduces fatty acid levels in plasma via the inhibition of lipolysis and induction of lipogenesis in adipose tissue and suppression of fatty acid production in liver (3). PA is a fungicide and bactericide and is therefore used as a food preservative (4, 5). It is also used to control fungi and bacteria growth in stored grains, hay, grain storage areas, poultry litter and drinking water (6). Pharmaceutical companies commonly include PA in the formulation of steroidal and nonsteroidal anti-inflammatory medi-cations. Fluticasone inhalers used for respiratory conditions, antihistamine and decongestant, comm-only contain PA.

PA derived from colonic bacterial fermen-tation contributes substantially to overall propionate load in children with disorders of propionate metabolism.The gut microbiota and its metabolites such as PA can access the brain through various routes within the liver – gut– brain axis (7, 8).The gut and the liver are intimately associated, and there is continuous bidirectional communication between these organs through the bile, hormones, inflammatory mediators, and the products of digestion and absorption. In the colon, PA is the metabolic end product of enteric bacteria, produced by fermentation of polysaccharides, oligosaccha-rides, long- chain fatty acids, proteins, peptides and glycoprotein precursors (9). The majority of the PA produced in the colon is absorbed, passes the colonocytes and the viscera, and drains into the portal vein. Around 90% of PA is metabolized by the liver and the rest is transported into the peripheral blood (10). Propionate affects various metabolic processes such as gluconeogenesis, ureogenesis or ketogenesis (11). However, there are also findings which appear inconsistent with the physiological role of propionate in the control of carbohydrate or lipid synthesis (12). PA is mainly metabolized in the liver and has been shown to inhibit gluconeogenis and increases glycolysis in rat hepatocytes (13). It has also been proposed that propionic acid may lower plasma cholesterol concentrations by inhibiting hepatic cholestero-genesis (14). PA influences the production of hormones through adipose tissue, such as the induction of leptin, which is a potent anorexi-genic hormone and suppresses food intake through receptors expressed in the central nervous system (15).

Even though PA is necessary for normal immune and physiological functioning; elevated levels may result in disruptive effects.(16). PA can readily cross the gut- liver- blood- brain barriers and gain access to the central nervous system (17). In the brain, it can cross cell membranes and accumulate within cells, inducing intracellular acidification (18, 19) which may alter neurotrans-mitter releases and, ultimately, neuronal communi-cation and behavior (20, 21). In fact, propionic acidemia is a neurodevelopmental metabolic disorder characterized by elevated levels of PA that clinically resembles some aspects of autism (22).

Liver is among the most important organs involved in metabolic activities. The intracellular enzymes of the parenchyma of liver cells have an amazing capacity to perform diverse and complicated biochemical functions. An elevation in the levels of serum marker enzymes with oxidative stress is generally regarded as one of the most sensitive indexes of hepatic damage (23). PA is neurotoxic and its axis to brain is through liver (7, 8).This study attempted to investigate the possible role of neurotoxic dose of PA on liver injury in male rats as the majority of PA is metabolized by the liver.

## Materials and methods

**Animals**

Adult male western albino rats weighing 150-200 g purchased from the animal house of science college, King Saud University, Riyadh were used throughout this study. The animals were fed on standard pellet diet and water ad libitum. The animals were maintained in a controlled environ-ment under standard conditions of temperature and humidity with an alternating light- and– dark cycle. All the procedures described were reviewed and approved by the King Saud University Animal Ethics Committee.

**Dosage and treatment**

Rats were randomly divided into two groups with ten rats in each. The first group served as a control. On the eighth day, the animals of the second group given an oral dosage of PA at the dose of (250 mg/kg body weight/day for three days; n= ten) (24). On the third day of PA administration, the rats were scarified and the liver organ was isolated. 

**Sample preparation**

**Blood collection for estimation of AST, ALT and ALP**

The blood was collected from retro-orbital plexus without the use of anticoagulant. The blood was allowed to stand for 10 min before being centrifuged at 2000 rpm for 10 min to obtain serum for analysis of alanine aminotransferase (ALT), aspartate aminotransferase (AST) and alkaline phosphatase (ALP)

**Tissue preparation**

Liver tissue was washed in ice cold normal saline and homogenized in a homogenizing buffer (50 mM Tris- HCl, 1.15% KCl pH 7.4) using Teflon homogenizer. The homogenate was centrifuged at 4000 g for 20 minutes to remove debris and kept at−80 °C until further use. The supernatant was used for the estimation of glutathione, lipid oxidation, glutathione-S-transferase, lactate dehydrogenase, potassium, sodium and vitamin C.

**Biochemical analysis**

**Serum alanine **aminotransferase** (ALT)**

ALT was estimated by the method of Reitman and Frankel (25). Briefly, 0.5 ml of substrate (2 mM α-ketoglutarate, 0.2 M DL-alanine in 0.1M phosphate buffer pH 7.4) was incubated at 37^ o^C for 5 minutes. 0.1 ml of freshly prepared serum was added to the aliquot and again incubated at 37 ^o^C for 30 minutes. At the end of incubation, 0.5 ml of 2, 4-dinitrophenyl hydrazine was added, and the aliquot left for 30 minutes at room temperature. 0.5 ml of 0.4 N NaOH was added, and the aliquot was again left for 30 minutes. Absorbance was then recorded at 505 nm against water blank.

**Serum aspartate aminotransferase (AST)**

AST was estimated by the method of Reitman and Frankel (25). The substrate, however, was 2 mM α-ketoglutarate, 0.2 M DL-aspartate, and the rest of the procedure was similar to ALT measurement method**.**

**Serum alkaline phosphatase (ALP)**

Serum alkaline phosphatase activity was measured according to the method of King and Armstrong (26), using disodium phenyl phosphate as substrate. The colour developed was read at 510 nm.

**Measurement of lipid peroxidation**

Lipid oxidation was evaluated by measuring the levels of lipid peroxidation by-products as thiobarbituric acid reactive substances (TBARS), namely malondialdehyde (MD), using the method of Ruiz-Larrea et al. (27). Accordingly, the samples were heated with TBA at low pH and the formation of a pink chromogen was measured by absorbance at 532 nm. The concentration of lipid peroxides was calculated as μ moles/ml using the extinction coefficient of MD.

**Assay of vitamin C**

Assay of vitamin C was performed according to the method of Jagota and Dani (28). 0.2 ml of liver homogenate was mixed with 0.8 ml of 10% trichloroacetic acid (TCA) and incubated in ice for 5 minutes. The samples were then centrifuged for 10 minutes at 3500 rpm and 4°C. 1.5 ml double distilled water was subsequently added to 0.5 ml of the supernatant. Eventually, 2 ml of Folin-phenol reagent was added and absorbance was measured at 760 nm after 10 minutes.

**Assay of glutathione (GSH)**

GSH content was determined according to the method described by Beutler et al. (29) using 5,5′-dithiobis 2-nitrobenzoic acid (DTNB) with sulfhy-dryl compounds to produce a relatively stable yellow color.

**Glutathione-S-transferase (GST) assay**

The GST activity was determined by the method of Habig et al. (30). The 1-chloro-2-4-di-nitrobenzene (CDNB) is neutralized by the enzyme in the presence of GSH as a cosubstrate. The change in absorbance is measured at 340 nm and the activity is expressed as nmol/min/mg protein.

**Assay of lactate dehydrogenase (LDH)**

The quantitative determination of LDH in the brain homogenates was performed using the lactate-to-pyruvate kinetic method described by Henry et al. (31).

**Determination of potassium levels**

Potassium levels were measured in a protein-

free alkaline medium by reaction with sodium tetraphenyl boron, which produced a colloidal suspension. The turbidity of such a suspension is proportional to the potassium concentrations in the range of 2–7 mmol/ l (32).

**Determination of sodium levels**

Sodium levels were assayed by enzymatic determination of sodium, i.e., the measurement of sodium-dependent galactosidase activity using ortho-Nitrophenyl- β- galactoside (ONPG) as a substrate (33)

**Statistical analysis**

The values are expressed as mean ± standard error of the mean (SEM). The results were evaluated using the SPSS (version 12.0) and Origin 6 softwares and evaluated by One -way ANOVA complemented with the Dunnett’s test for multiple comparisons. Statistical significance was considered when The p-value was < 0.05

## Results

**Effect of PA on hepatic markers**

The activities of AST, ALT and ALP are presented in Table 1. We observed statistically a significant increase in AST and ALP in serum of rats exposed to PA (250 mg/kg body weight/day for three days). Also the administration of PA induced a marked increase in ALT levels as compared to the control group.

**Effect of PA on GSH and antioxidant enzyme activities**

Table 2 presents the mean ± SEM of the GSH (μg/ml), MD (μmoles/ml) and vitamin C (μg/ml) concentrations, GST (U/ml) and LDH activities in the liver homogenates of the two groups of rats. Compared to control groups, the PA-treated rats exhibited a statistically significant increase in MD and LDH activities with a concomitant decrease of GST, GSH, and Vitamin C.

**Table 1:** Serum AST, ALT and ALP activities

**Parameters**		**Groups**	**Min**		**Max**		**Mean ± SEM**	**P value**
AST (U/L)		Control		139.67	154.7		147.62±3.38		0.01
		PA		203.32	365.09		247.34±30.09		
ALT (U/L)		Control		52.45	104.31		84.27±9.17		0.09
		PA		98.41	174.44		117.03±14.43		
ALP (U/L)		Control		365.7	615.48		496.24±43.24		0.05
		PA		533.37	862.5		664.884±61.31		

AST: serum aspartate aminotransferase; ALT: serum alanine aminotransferase; ALP: serum alkaline phosphatase;.max: maximum; min: minimum; PA: propionic acid; SEM: standard error of the mean.

**Table 2:** GSH, MD, vitamin C concentrations and GST and LDH activities in the liver homogenates

**Parameters**	**Groups**	**Min**	**Max**	**Mean± SEM**	**P value**
GSH(μg /ml)	Control	57.97	79.71	66.18±4.62	0.01
	PA	24.15	50.72	45.40±5.31	
MD (μmoles /ml)	Control	0.113	0.151	0.13±0.007	0.01
	PA	0.16	0.33	0.23±0.030	
Vitamin C (μg /ml)	Control	39.4	100	68.75±11.09	0.04
	PA	37.8	39.4	38.48±0.32	
GST (U /ml)	Control	38.75	54.27	48.24±3.05	0.05
	PA	3.08	40.83	30.54±7.09	
LDH (U /L)	Control	130.43	161.83	148.77±5.43	0.001
	PA	185.99	239.13	208.2±10.78	

GSH: glutathione; MD: malondialdehyde; LDH: lactate dehydrogenase; max: maximum; min: minimum; SEM: standard error of the mean.

**Table 3:** Sodium and potassium levels in the liver homogenates

**Parameters**	**Groups**	**Min**	**Max**	**Mean ± SEM**	**P value**
Potassium mmol/L	Control	7.6	8.48	8.05±0.69	0.06
	PA	8.22	8.93	8.52±0.45	
Sodium mmol/L	Control	114.3	118.10	116.53±0.16	0.62
	PA	115.1	117.3	116.94±0.12	

max: maximum; min: minimum; SEM: standard error of the mean

**Effect of PA on sodium and potassium**

**Fig. 1:**
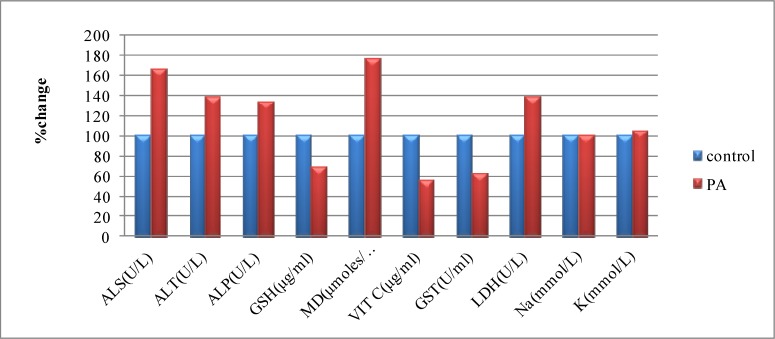
Percentage change of all parameters in PA group compared to control.

In liver tissue, sodium and potassium levels were not affected by PA treatment as shown in Table 3. Figure 1 shows the percentage change of all parameters in PA treated group compared to control.

## Discussion

The levels of some important biochemical parameters in serum are used as diagnostic markers of hepatic injury. One of the most sensitive and dramatic indicators of hepatocyte injury is the release of intracellular enzymes, such as transaminases and serum alkaline phosphatase. The elevated activities of these enzymes are indicative of cellular leakage and the loss of the functional integrity of the cell membranes in liver which are always associated with hepatonecrosis (34-36). The oral administration of PA showed drastic alterations in the level of serum marker enzymes AST, ALT and ALP when compared with control rats as shown in Table 1, indicating PA mediated hepatic damages. ALP is a membrane associated enzyme and an increased activity of ALP is an indication of liver damage (37). ALT is more abundant in the liver cells than in any other cells in the body and is primarily used as a specific marker of hepatic damage. ALT and AST enzymes are regarded as markers of liver injury since liver is the major site of metabolism (38, 39).

Liver plays a pivotal role in the regulation of various physiological processes in the body such as carbohydrate metabolism and storage, fat metaboli-sm, bile acid synthesis, and so forth besides being the most important organ involved in the detoxification of various drugs as well as xenobiotics in our body (40). Antioxidant enzymes act in a coordinated fashion to prevent the oxidative stress. Thiobarbituric acid reactive substances (TBARS), the final metabolites of peroxidized polyunsaturated fatty acids and the late biomarker of oxidative stress (41) were significantly increased in our results, providing a direct assessment of the progression of liver injury at the cellular level (42).The increased level of LDH in PA treated group is an indication of abnormality in liver functioning, which may be due to the formation of highly reactive free radicals due to PA treatment. Reduced glutathione (GSH) is an important reductant in the cell, where it protects against free radicals, peroxides and other toxic components. It maintains the normal structure and function of cells, probably by its redox and detoxification reactions (43). The decreased concentration of GSH in liver tissue found in PA treated group of our study may be due to NADPH reduction or GSH utilization in the exclusion of peroxides (44). Support for this comes from some previous findings (21, 45) in which the high levels of PA were reported to induce oxidative stress with decreased levels of total GSH in brain tissue.

Circulating antioxidants such as vitamin C are non-enzymatic scavengers of free radicals. A decrease in ascorbic acid levels in plasma (46) and liver (47) was reported in injured liver in rats. Decreased levels of vitamin C found in PA treated rats in the present study are in line with the findings of a previous study by El-Ansary et al. (45).

Glutathione-S-transferase is actually compos-ed of a group of isoenzymes capable of detoxifying various exogenous and endogenous substances by conjugation with glutathione. A reduction in the activity of these enzymes is associated with the accumulation of highly reactive free radicals, leading to deleterious effects such as loss of integrity and function of cell membranes (48, 49). Significant decrease of GST reported in the present study could easily be related to the oxidative effect of PA previously reported by Alfawaz et al. (50).

The active transport of sodium- potassium across the cell membrane is controlled by sodium- potassium-adenosine triphosphatase (Na+-K+-ATPase) enzyme, which is an integral plasma membrane protein responsible for a large part of the energy consumption constituting the cellular metabolic rate. Na+-K+-ATPase controls cell volume, nerve and muscle signals and drives the transport of amino acids and sugars (51). However, the non significant difference from the control value by administration of PA suggests that it may be of low toxicity to the monovalent ion.

In conclusion, increased activities of AST, ALT, and ALP suggest severe hepatic injury resulting from the administration of PA. The increase of TBARS in liver of treated rats provides evidence for the pathogenic role of PA in inducing oxidative liver injury.
